# Association between mass media exposure and HIV testing uptake in Cameroon

**DOI:** 10.1371/journal.pgph.0003687

**Published:** 2024-09-12

**Authors:** Roger Antabe, Yujiro Sano, Daniel Amoak

**Affiliations:** 1 Department of Health and Society, University of Toronto Scarborough, Toronto, Onatrio, Canada; 2 Department of Sociology and Anthropology, Nipissing University, North Bay, Onatrio, Canada; 3 Department of Geography and Environment, Western University, London, Onatrio, Canada; The Chinese University of Hong Kong, HONG KONG

## Abstract

In sub-Saharan African countries, mass media is critical in disseminating health information, including the need for HIV testing. Yet, in Cameroon, there is a dearth of studies examining how exposure to mass media is effective in the uptake of HIV testing. Using the 2018 Cameroon Demographic and Health Survey, we examined the association between exposure to mass media and HIV testing among sexually active women (n = 12,619) and men (n = 5,607). Our findings revealed a generally low uptake of HIV testing although more women (78%) have ever tested for HIV compared to men (67%). Adjusting for demographic, socioeconomic, and psychosocial factors, we found for both women and men their exposure at least once a week to the Internet (aOR = 1.57, p<0.001 for women; aOR = 1.76, p<0.001 for men), print media (aOR = 1.59, p<0.05 for women; aOR = 2.04, p<0.001 for men), radio (aOR = 1.34, p<0.01 for women; aOR = 1.57, p<0.001 for men), and television (aOR = 1.74, p<0.001 for women; aOR = 1.94, p<0.001 for men) was significantly associated with a higher likelihood of testing for HIV compared to their counterparts with no exposure at all. Our findings underscore the importance of further integrating mass media in HIV messaging in Cameroon as the country aims to achieve UNAIDS target 95-95-95 by 2023.

## Introduction

Cameroon significantly reduced HIV prevalence from 5.4% in 2004 to 2.7% in 2018. Recent data from the Joint United Nations Programme on HIV/AIDS (UNAIDS) suggest that this may have further declined to 2.6% in 2022 [1, 2]. Cameroon is one of the only two countries (the other being Zimbabwe) in sub-Saharan Africa (SSA) commended by UNAIDS for drastically reducing HIV prevalence and new HIV infections through its national prevention programs [3]. Despite this significant progress, the country has the highest HIV prevalence rate in West Central Africa, and globally, it ranks 15^th^ among countries with the highest HIV prevalence [4, 5]. These statistics have raised critical concerns about Cameroon’s ability to meet the UNAIDS target of 95-95-95 by 2030, where 95% of people living with HIV know their HIV, 95% of those living with HIV are on antiretroviral treatment, and 95% of those on antiretroviral treatment have achieved viral suppression [3, 5]. A 2022 estimate suggested these rates stood at 95.8%, 92.3% and 89.2% [6].

Earlier studies in Cameroon have suggested a generally low uptake of HIV testing among the population. For instance, a 2020 report showed that only 65.9% of females and 52.5% of males aged 15–64 years have ever tested for HIV and received results for their diagnoses. The report further revealed that among Persons Living with HIV (PLHIV) in the country, 49.4% of females and 41.2% of males reported knowing their status [7]. Additionally, the reported incidence rate ranges from 0.24% to 0.27% in 2017–2018 [7, 8]. According to the U.S President’s Emergency Fund for AIDS Relief (PEPFAR), an estimated 14,600 new infections occurred in 2020, with a total of 504,0000 PLHIV in the country [9]. Addressing the high HIV incidence and prevalence rates and creating access to HIV services may be financially daunting for the government of Cameroon. For instance, it is indicated that for the 2018–2019 period, the National AIDS Control Committee (NACC) in the country was only able to raise 37% of the expected funding for its HIV prevention strategies and activities, leaving a funding gap of 63% [10]. Similarly, in 2017, out of the total $130 million funding budget for HIV response in Cameroon, only a quarter (25.4%) was provided by the government, with the majority of the funding coming from other sources (5.4%) and through development assistance (69.2%) [9]. Amidst a declining budgetary allocation to the Ministry of Health over the years, it is estimated that about 70% of the cost related to accessing HIV services in the country is taken up by the household [11–13]. In unpacking the driving forces for the uptake of HIV testing in Cameroon, evidence has pointed to the influence of several factors. These may include the role of gender where men feel health care spaces providing HIV testing services are reserved for women [14], stigma, discrimination and secrecy related to the disclosure of an HIV-positive status after testing [15], region and rural-urban residency which define the ease of access to HIV related resources including information and testing services [16], and socioeconomic status where Cameroonians with higher levels of educational attainment and household wealth are more likely to have ever tested for HIV [7].

Over the years in Cameroon, the emphasis has been among others, to intensify HIV information dissemination to the population using a variety of means, particularly mass media platforms as a way to increase the uptake of HIV testing given its crucial role as the first step to the HIV care cascade [17]. For instance, the first National AIDS Control Programme (NACP) in 1987, which ran in two phases from 1988 to 1995 and then the first (2000–2005) and second (2006–2010) National AIDS Strategic Plans recommended the use of mass media, and improving partnership with the mediascape in order to ease the dissemination of HIV information as part of the national HIV prevention strategies [18, 19]. Other key stakeholders in the fight against HIV in the country, including non-governmental organizations (NGOs), also employ the use of mass and social media platforms to, among others, create awareness about HIV, reduce HIV misconceptions and stigma, and encourage the uptake of HIV testing. In its 2022 strategic plan for Cameron for example, the United States President’s Emergency Plan for AIDS Relief (PEPFAR) alluded to the need to use mass media platforms to reach adolescents girls and young women, boys and younger men and other ‘at risk’ groups as they are more vulnerable to new HIV infection [20].

Muluh and Nukwa [21] and Wantchami [22] had earlier called for the mass media in Cameroon to be more proactive in the country’s fight against HIV. The authors proposed the mass media use their respective platforms to engage the public in mass education on HIV transmission and working to normalize HIV testing among citizens. While such calls may be relevant, there is uncertainty about how people’s mass media exposure in Cameroon is associated with the uptake of HIV testing. It is concerning that although mass media continues to play an important role in HIV prevention strategies in the country, there is a dearth of studies exploring how people’s exposure to types of mass media is associated with HIV testing uptake. Understanding the relationship between the type of media exposure and HIV testing is critical for Cameroon to meet UNAIDS’ 95-95-95 target as the country strategizes to effectively target citizens with health information that encourages HIV testing and treatment.

The utility of using mass media platforms to disseminate health information, including HIV testing, in the settings of SSA, including Cameroon, may be hinged on several factors. First, the use of mass media in this context has been shown to be associated with several positive health outcomes. For instance, in Ghana, exposure to mass media was observed to be associated with the uptake of HIV testing. Specifically, higher exposure to radio among women and print and television among men was associated with the uptake of HIV testing [23]. In Kenya, Onsomu et al. [24] revealed the importance of the mass media in scaling up HIV testing in the country, observing that exposure to newspapers/magazines among women and television among men was associated with testing for HIV. In a cross-sectional study of three countries in SSA, it was established that, except for Zambia, there was an increase in the number of youths in Nigeria and Kenya testing for HIV due to their exposure to mass media [25]. Additionally, mass media is seen to be effective in improving people’s HIV prevention behaviours, such as the use of condoms and boosting HIV transmission knowledge [26]. Furthermore, mass media exposure is observed to increase the uptake of modern contraceptives among postpartum women, the use of antenatal care services among women, improving household decision-making autonomy of women, and enrolment into health insurance [27–31]. Even where the mass media does not directly disseminate messages on HIV, it may still positively influence the uptake of HIV testing. This is because exposure to mass media is known to increase general health knowledge and further improve health information literacy [32, 33]. Improved general health knowledge and health information literacy as desirable outcomes are noted to be associated with positive health behaviour changes such as the uptake of HIV testing [34]. Second, the healthcare system and healthcare infrastructure in most areas of SSA, which serve as sources of health information, are often in a deplorable state and may not be well-resourced to provide these services. These facilities tend to lack adequate staffing and healthcare professionals with the requisite expertise in health promotion and education to serve their operational communities [35, 36]. Third, most healthcare systems in SSA are curative and may not have fully functional health information units that engage the public in preventive health care, including providing information on HIV prevention strategies and HIV testing [37].

In addition, healthcare infrastructure and resources in SSA may not be evenly distributed. There is an urban bias in the allocation of healthcare infrastructure that leaves most rural areas lacking in their access to basic healthcare services [38, 39]. Even where health information is to be delivered by trained healthcare professionals through outreach programs, geographic barriers, including the remoteness of some communities and the unmotorable nature of their roads make it difficult to access them. In some cases, these communities become completely cut off during the rainy season making it particularly challenging to reach residents with health services or information [30, 40]. Finally, there has been extensive progress in the deployment of mass media in SSA within the last few decades which has opened opportunities for reaching many people through mass communication. This expansion has made mass media cheaper but an effective medium to reach the population including those in rural and remote areas with critical health information [30, 36]. To this end, the purpose of this study was to examine how exposure to different types of mass media is associated with the uptake of HIV testing in Cameroon.

## Methods

### Data source

Descriptive cross-sectional design was used for this study. We used the 2018 Cameroon Demographic and Health Survey (CDHS) to explore the relationship between the uptake of HIV testing and mass media exposure in Cameroon. The 2018 CDHS is a nationally representative survey, which was implemented by the National Institute of Statistics in collaboration with the Ministry of Public Health. The CDHS utilized a multi-staged sampling framework in which systematic sampling with probability to size was applied to identify enumeration areas from which households were chosen. Originally, 14,677 women and 6,978 men aged 15 to 64 years were included as part of the sample, with a response rate of 98% for both women and men. As we focused on sexually active respondents, we excluded 2,058 women and 1,371 men who have never had sexual intercourse from our analyses. This further restricted the analytical sample to sexually active respondents, leading to our final sample of 12,619 women and 5,607 men. More information about the study design, methodology and survey questionnaires have been described elsewhere [41].

### Measures

#### Outcome variable

The outcome variable for this study is HIV Testing Uptake. All sexually active respondents were asked if they have ever tested for HIV. Using this question, we have constructed the dependent variable called ‘ever tested for HIV’ (0 = no; 1 = yes).

#### Independent and control variables

Four main independent variables include frequency of exposure to the Internet, print media, television, and radio (0 = not at all; 1 = less than once a week; 2 = at least once a week). Informed by a review of the literature on the factors that could impact the uptake of HIV testing [42–44], we introduced three sets of control variables, namely socioeconomic, demographic, and psychosocial variables. There were three socioeconomic variables including education (0 = no education; 1 = primary education; 2 = secondary/higher education), household wealth (0 = poorest; 1 = poorer; 2 = middle; 3 = richer; 4 = richest), and employment status (0 = unemployed; 1 = employed). Two demographic variables such as place of residence (0 = urban; 1 = rural) and age of respondents (measured in completed year) were also added. Finally, psychosocial factors such as adequate HIV knowledge (0 = no; 1 = yes) and HIV stigma (0 = yes; 1 = no) were also integrated in statistical analysis.

### Statistical analysis

We employed three separate analyses for this study. First, descriptive analysis was performed, capturing the characteristics of our analytical sample. In addition, unadjusted analyses were employed for understanding the gross relationships between the dependent and independent/control variables. We further employed adjusted analyses to simultaneously account for independent and control variables, which enabled us to estimate net impacts. In terms of adjusted analyses, we built eight models–four each for women and men. Each model included the frequency of using the Internet, reading newspaper/magazine, listening to radio, and watching television as the focal independent variable. For unadjusted and adjusted analyses, we relied on logistic regression analysis due to the dichotomous nature of the dependent variable. Results are shown with odds ratios (ORs). All analyses used STATA 17 (State Corp, College Station, TX, USA). The ‘svy’ function was applied in statistical analysis to adjust for the cluster sampling design as well as sampling weights [45].

### Ethical consideration

Our study used a secondary dataset with no identifiable information on study participants. The authors were given permission by the Demographic and Health Survey program to use the dataset. More about this survey can be found elsewhere [41]. The data can be assessed at: https://dhsprogram.com/data/dataset/Cameroon_StandardDHS_2018.cfm?flag=0

## Results

Table 1 shows sample characteristics. We found that 78% (9,843) and 67% (3,694) of women and men have ever been tested for HIV, respectively. In addition, the proportion of those who were exposed to mass media once a week was largest for television (43% [5,477] and 48% [2,684] for women and men, respectively), followed by the Internet (22% [2,772] and 33% [1,811]), radio (19% [2,308] and 33% [1,829]), and print media (6% [767] and 12% [659]). Turning into socioeconomic characteristics, 47% (5,952) and 62% (3,394) of women and men had secondary/higher education, respectively. We also found that 68% (8,510) and 88% (4,863) of women and men were employed, respectively. In terms of demographic characteristics, slightly less than half of women (47% [5,837]) and men (44% [2,414]) lived in rural areas. When it comes to psychosocial characteristics, 71% (8,923) of women and 73% (4,065) of men have adequate HIV knowledge while 57% (7,147) and 54% (2,985) of women and men did not have any HIV stigma, respectively.

**Table 1:** Distribution of selected background characteristics of sexually active women and men (15–64 years), 2018 CDHS.

	Women (n = 12,563)		Men (n = 5,549)	
	Percentage (95% CI)	Weighted counts	Percentage (95% CI)	Weighted counts
**Ever tested for HIV**				
No	22 (20, 23)	2,721	33 (31, 35)	1,855
Yes	78 (77, 80)	9,843	67 (65, 69)	3,694
**Frequency of using the Internet**				
Not at all	75 (73, 76)	9,366	61 (59, 64)	3,403
Less than once a week	3 (3, 4)	426	6 (5, 7)	334
At least once a week	22 (20, 24)	2,772	33 (31, 35)	1,811
**Frequency of reading newspaper or magazine**				
Not at all	81 (80, 83)	10,209	71 (69, 73)	3,932
Less than once a week	13 (12, 14)	1,587	17 (16, 19)	958
At least once a week	6 (5, 7)	767	12 (11, 13)	659
**Frequency of listening to radio**				
Not at all	61 (60, 63)	7,711	42 (40, 44)	2,332
Less than once a week	20 (19, 22)	2,544	25 (23, 27)	1,387
At least once a week	19 (17, 20)	2,308	33 (31, 35)	1,829
**Frequency of watching television**				
Not at all	45 (42, 48)	5,634	36 (33, 39)	1,991
Less than once a week	12 (11, 13)	1,452	16 (14, 18)	875
At least once a week	43 (41, 46)	5,477	48 (45, 51)	2,684
**Education**				
No education	24 (22, 26)	3,007	12 (10, 14)	671
Primary education	29 (27, 30)	3,604	26 (25, 29)	1,483
Secondary/higher education	47 (45, 50)	5,952	62 (59, 63)	3,394
**Household wealth**				
Poorest	17 (15, 19)	2,113	13 (11, 15)	740
Poorer	19 (17, 22)	2,453	18 (16, 20)	1,004
Middle	20 (19, 22)	2,555	21 (19, 23)	1,153
Richer	21 (20, 23)	2,668	22 (20, 24)	1,225
Richest	22 (20, 25)	2,792	26 (23, 28)	1,428
**Employment status**				
Unemployed	32 (31, 34)	4,053	12 (11, 14)	686
Employed	68 (66, 69)	8,510	88 (86, 89)	4,863
**Place of residence**				
Urban	53 (51, 56)	6,726	56 (53, 59)	3,135
Rural	47 (44, 49)	5,837	44 (41, 47)	2,414
**Age of respondents**				
15–19	12 (11, 13)	1,464	8 (7, 9)	452
20–24	17 (17, 18)	2,207	16 (15, 17)	882
25–29	19 (18, 20)	2,391	16 (15, 17)	895
30–34	16 (15, 16)	1,958	14 (13, 15)	785
35–39	12 (11, 13)	1,492	12 (11, 13)	675
40–44	9 (8, 9)	1,088	10 (9, 12)	576
45–49	7 (7, 8)	902	8 (7, 9)	432
50+	8 (8, 9)	1,061	15 (14, 16)	851
**Adequate HIV knowledge**				
No	29 (27, 31)	2,640	27 (25, 29)	1,484
Yes	71 (69, 73)	8,923	73 (71, 75)	4,065
**HIV stigma**				
Yes	43 (41, 45)	5,417	46 (44, 49)	2,564
No	57 (55, 59)	7,147	54 (51, 56)	2,985

Table 2 shows unadjusted findings from regression analysis for women and men. We found that women and men who were exposed to the Internet less than once a week (uOR = 3.26, 95% CI = 2.14, 4.96; uOR = 2.63, 95% CI = 1.82, 3.79 for women and men) and at least once a week (uOR = 6.90, 95% CI = 5.52, 8.63; uOR = 3.59, 95% CI = 2.99, 4.32 for women and men) were more likely to have been tested for HIV compared to those who were not exposed at all. Similarly, we found that women and men who were exposed to print media less than once a week (uOR = 4.40, 95% CI = 3.54, 5.48; uOR = 3.51, 95% CI = 2.82, 4.38 for women and men) and at least once a week (uOR = 7.24; 95% CI = 4.92, 10.63; uOR = 5.62, 95% CI = 4.19, 7.54 for women and men) were more likely to have been tested for HIV compared to those who were not exposed at all. Consistent with the Internet and print media, we found that women and men who were exposed to radio less than once a week (uOR = 2.89, 95% CI = 2.41, 3.47; uOR = 2.74, 95% CI = 2.26, 3.31 for women and men) and at least once a week (uOR = 3.94; 95% CI = 3.30, 4.70; uOR = 3.97, 95% CI = 3.29, 4.80 for women and men) were more likely to have been tested for HIV compared to those who were not exposed at all. We also found that women and men who were exposed to television less than once a week (uOR = 3.26, 95% CI = 2.65, 4.00; uOR = 3.01, 95% CI = 2.35, 3.86 for women and men) and at least once a week (uOR = 6.32; 95% CI = 5.39, 7.40; uOR = 4.93, 95% CI = 4.03, 6.01 for women and men) were more likely to have been tested for HIV compared to those who were not exposed at all.

**Table 2:** Unadjusted logistic regression models predicting ‘ever tested for HIV’ among sexually active women and men.

	Women			Men		
	uOR	95% CI		uOR	95% CI	
**Frequency of using the Internet**						
Not at all	1.00			1.00		
Less than once a week	3.26***	2.14	4.96	2.63***	1.82	3.79
At least once a week	6.90***	5.52	8.63	3.59***	2.99	4.32
**Frequency of reading newspaper or magazine**						
Not at all	1.00			1.00		
Less than once a week	4.40***	3.54	5.48	3.51***	2.82	4.38
At least once a week	7.24***	4.92	10.63	5.62***	4.19	7.54
**Frequency of listening to radio**						
Not at all	1.00			1.00		
Less than once a week	2.89***	2.41	3.47	2.74***	2.26	3.31
At least once a week	3.94***	3.30	4.70	3.97***	3.29	4.80
**Frequency of watching television**						
Not at all	1.00			1.00		
Less than once a week	3.26***	2.65	4.00	3.01***	2.35	3.86
At least once a week	6.32***	5.39	7.40	4.93***	4.03	6.01
**Education**						
No education	1.00			1.00		
Primary education	4.09***	3.44	4.87	5.15***	3.91	6.78
Secondary/higher education	10.06***	8.35	12.13	10.10***	7.70	13.25
**Household wealth**						
Poorest	1.00			1.00		
Poorer	2.57***	2.07	3.19	2.85***	2.23	3.64
Middle	4.17***	3.35	5.20	4.23***	3.25	5.51
Richer	8.05***	6.28	10.31	7.20***	5.45	9.52
Richest	14.89***	11.18	19.83	13.88***	10.34	18.62
**Employment status**						
Unemployed	1.00			1.00		
Employed	1.18*	1.01	1.38	0.97	0.77	1.23
**Place of residence**						
Urban	1.00			1.00		
Rural	0.30***	0.25	0.35	0.36***	0.30	0.43
**Age of respondents**						
15–19	1.00			1.00		
20–24	2.50***	2.08	3.01	3.18***	2.46	4.12
25–29	3.11***	2.55	3.79	4.13***	3.10	5.50
30–34	3.27***	2.62	4.07	4.45***	3.33	5.95
35–39	2.61***	2.13	3.20	4.20***	3.03	5.82
40–44	2.42***	1.96	2.99	4.01***	2.93	5.49
45–49	1.50**	1.16	1.93	3.74***	2.60	5.38
50+	1.14	0.92		3.04***	2.34	3.94
**Adequate HIV knowledge**						
No	1.00			1.00		
Yes	2.93***	2.51	3.41	3.11***	2.63	3.67
**HIV stigma**						
Yes	1.00			1.00		
No	3.80***	3.29	4.40	2.55***	2.18	2.98

uOR = odds ratio; CI = confidence intervals

*p<0.05

**p<0.01

***p<0.001

Tables 3 and 4 show findings from adjusted regression analyses for women and men, respectively. Findings were largely consistent with unadjusted findings, even after accounting for socioeconomic, demographic, and psychosocial factors. We found that men who were exposed to the Internet less than once a week (aOR = 1.77, 95% CI = 1.15, 2.71) and women and men exposed to the Internet at least once a week (aOR = 1.57, 95% CI = 1.22, 2.00; aOR = 1.76, 95% CI = 1.41, 2.21 for women and men) were more likely to have been tested for HIV compared to those who were not exposed at all. Similarly, we found that women and men who were exposed to print media less than once a week (aOR = 1.37, 95% CI = 1.10, 1.70; aOR = 1.60, 95% CI = 1.28, 2.00 for women and men) and at least once a week (aOR = 1.59; 95% CI = 1.07, 2.35; aOR = 2.04, 95% CI = 1.50, 2.78 for women and men) were more likely to have been tested for HIV compared to those who were not exposed at all. Consistent with the Internet and print media, we found that women and men who were exposed to radio less than once a week (aOR = 1.33, 95% CI = 1.11, 1.59; aOR = 1.42, 95% CI = 1.16, 1.73 for women and men) and at least once a week (aOR = 1.34; 95% CI = 1.11, 1.61; aOR = 1.57, 95% CI = 1.30, 1.90 for women and men) were more likely to have been tested for HIV compared to those who were not exposed at all. We also found that women and men who were exposed to television less than once a week (aOR = 1.39, 95% CI = 1.13, 1.72; aOR = 1.60, 95% CI = 1.26, 2.03 for women and men) and at least once a week (aOR = 1.73, 95% CI = 1.44, 2.09; aOR = 1.94, 95% CI = 1.52, 2.46 for women and men) were more likely to have been tested for HIV compared to those who were not exposed at all.

**Table 3:** Adjusted logistic regression models predicting ‘ever tested for HIV’ among sexually active women in Cameroon.

	The Internet			Newspaper/magazine			Radio			Television		
	aOR	95% CI		aOR	95% CI		aOR	95% CI		aOR	95% CI	
**Frequency of using mass media**												
Not at all	1.00			1.00			1.00			1.00		
Less than once a week	1.01	0.68	1.52	1.36**	1.10	1.70	1.32**	1.11	1.58	1.40**	1.13	1.73
At least once a week	1.57***	1.22	2.00	1.59*	1.07	2.36	1.34**	1.11	1.61	1.74***	1.44	2.09
**Education**												
No education	1.00			1.00			1.00			1.00		
Primary education	2.58***	2.15	3.10	2.53***	2.10	3.05	2.47***	2.05	2.98	2.41***	2.00	2.90
Secondary/higher education	4.25***	3.41	5.29	4.28***	3.42	5.35	4.25***	3.41	5.30	3.96***	3.18	4.93
**Household wealth**												
Poorest	1.00			1.00			1.00			1.00		
Poorer	1.83***	1.45	2.31	1.83***	1.45	2.31	1.79***	1.42	2.25	1.79***	1.42	2.26
Middle	2.28***	1.77	2.94	2.26***	1.76	2.92	2.15***	1.66	2.79	1.99***	1.54	2.57
Richer	3.23***	2.29	4.55	3.30***	2.33	4.67	3.12***	2.19	4.44	2.49***	1.73	3.56
Richest	3.73***	2.48	5.61	4.07***	2.73	6.07	3.94***	2.63	5.92	3.05***	2.01	4.64
**Employment status**												
Unemployed	1.00			1.00			1.00			1.00		
Employed	1.37***	1.15	1.64	1.37***	1.14	1.64	1.36***	1.13	1.63	1.38***	1.15	1.65
**Place of residence**												
Urban	1.00			1.00			1.00			1.00		
Rural	0.99	0.79	1.24	0.98	0.78	1.22	0.96	0.76	1.21	1.00	0.80	1.26
**Age of respondents**												
15–19	1.00			1.00			1.00			1.00		
20–24	2.48***	1.96	3.14	2.52***	1.99	3.20	2.53***	1.99	3.20	2.54***	2.01	3.21
25–29	4.23***	3.30	5.41	4.27***	3.34	5.46	4.28***	3.35	5.48	4.30***	3.37	5.49
30–34	4.67***	3.64	6.00	4.71***	3.67	6.05	4.69***	3.65	6.02	4.75***	3.70	6.10
35–39	3.74***	2.91	4.81	3.76***	2.93	4.83	3.74***	2.90	4.81	3.79***	2.95	4.87
40–44	3.48***	2.68	4.53	3.45***	2.65	4.49	3.45***	2.65	4.49	3.49***	2.67	4.55
45–49	2.01***	1.45	2.77	1.97***	1.43	2.72	1.96***	1.41	2.71	2.02***	1.46	2.79
50+	1.96***	1.49	2.59	1.94***	1.47	2.56	1.93***	1.46	2.34	1.96***	1.49	2.59
**Adequate HIV knowledge**												
No	1.00			1.00			1.00			1.00		
Yes	1.83***	1.55	2.17	1.84***	1.55	2.18	1.84***	1.55	2.19	1.85***	1.56	2.19
**HIV stigma**												
Yes	1.00			1.00			1.00			1.00		
No	1.93***	1.63	2.29	1.93***	1.63	2.29	1.94***	1.63	2.30	1.93***	1.63	2.29

aOR = adjusted odds ratio; CI = confidence intervals

*p<0.05

**p<0.01

***p<0.001

**Table 4:** Adjusted logistic regression models predicting ‘ever tested for HIV’ among sexually active men in Cameroon.

	The Internet			Newspaper/magazine			Radio			Television		
	aOR	95% CI		aOR	95% CI		aOR	95% CI		aOR	95% CI	
**Frequency of using mass media**												
Not at all	1.00			1.00			1.00			1.00		
Less than once a week	1.76**	1.15	2.70	1.60***	1.28	2.00	1.42***	1.16	1.73	1.60***	1.26	2.03
At least once a week	1.76***	1.41	2.21	2.04***	1.50	2.78	1.57***	1.30	1.90	1.94***	1.52	2.47
**Education**												
No education	1.00			1.00			1.00			1.00		
Primary education	3.71***	2.82	4.89	3.54***	2.68	4.66	3.41***	2.57	4.51	3.39***	2.56	4.48
Secondary/higher education	5.22***	3.88	7.01	4.95***	3.65	6.71	4.99***	3.71	6.72	5.03***	3.73	6.79
**Household wealth**												
Poorest	1.00			1.00			1.00			1.00		
Poorer	2.02***	1.55	2.64	1.99***	1.52	2.60	1.90***	1.46	2.47	1.90***	1.45	2.48
Middle	2.71***	2.04	3.60	2.74***	2.07	3.65	2.53***	1.90	3.36	2.24***	1.69	2.97
Richer	3.70***	2.63	5.21	3.86***	2.75	5.42	3.64***	2.59	5.11	2.87***	2.04	4.04
Richest	5.90***	3.94	8.84	6.54***	4.39	9.76	6.28***	4.21	9.37	4.88***	3.31	7.20
**Employment status**												
Unemployed	1.00			1.00			1.00			1.00		
Employed	1.17	0.89	1.55	1.15	0.87	1.51	1.13	0.86	1.47	1.14	0.87	1.49
**Place of residence**												
Urban	1.00			1.00			1.00			1.00		
Rural	1.09	0.85	1.39	1.06	0.83	1.35	1.02	0.80	1.29	1.09	0.86	1.38
**Age of respondents**												
15–19	1.00			1.00			1.00			1.00		
20–24	3.71***	2.78	4.94	3.59***	2.69	4.77	3.65***	2.74	4.85	3.85***	2.88	5.14
25–29	7.15***	5.22	9.81	6.39***	4.68	8.73	6.68***	4.88	9.15	7.28***	5.33	9.95
30–34	9.05***	6.52	12.57	7.76***	5.63	10.71	8.04***	5.84	11.06	8.96***	6.48	12.37
35–39	9.58***	6.48	14.15	8.17***	5.52	12.12	8.45***	5.72	12.49	9.43***	6.40	13.91
40–44	10.00***	7.05	14.20	8.10***	5.76	11.38	8.26***	5.87	11.64	9.41***	6.66	13.30
45–49	10.14***	6.92	14.85	8.14***	5.55	11.95	8.21***	5.61	12.02	9.27***	6.33	13.57
50+	8.85***	6.49	12.06	6.85***	5.06	9.28	6.99***	5.17	9.45	8.06***	5.97	10.88
**Adequate HIV knowledge**												
No	1.00			1.00			1.00			1.00		
Yes	2.16***	1.79	2.60	2.19***	1.82	2.64	2.13***	1.77	2.55	2.13***	1.77	2.56
**HIV stigma**												
Yes	1.00			1.00			1.00			1.00		
No	1.53***	1.31	1.79	1.51***	1.29	1.77	1.53***	1.31	1.79	1.53***	1.31	1.80

aOR = adjusted odds ratio; CI = confidence intervals

*p<0.05

**p<0.01

***p<0.001

In addition to mass media exposure, we found a range of control variables associated with the uptake of HIV testing among women and men. For example, women and men with primary education and secondary/higher education were more likely to have been tested for HIV than those without any formal education. Similarly, women and men whose household wealth belongs to the ‘poorer’, ‘middle’, ‘richer’, and ‘richest’ category were more likely to have been tested for HIV compared to those belonging to the ‘poorest’ category. We also found that employed women and men were more likely to have been tested for HIV compared to their unemployed counterparts. In terms of age, we found that older women and men were more likely to have been tested for HIV compared to their youngest counterparts (i.e., 15–19), except women aged 60–64 did not significantly differ. Women and men with adequate HIV knowledge were more likely to have been tested for HIV compared to those without it. Finally, women and men without any HIV stigma were more likely to have been tested for HIV compared to those who had some HIV stigma.

## Discussion

Over the years, Cameroon has made significant progress in reducing HIV prevalence from 6% in the 2000s to 2.6% in 2022 [1]. Regardless of this progress, the country has one of the highest HIV prevalence globally and is the most HIV endemic in the West Central African Region [5]. Given the potential of HIV campaign through mass media to positively influence HIV prevention behaviors, the country has employed its use in its national strategy to address the high levels of HIV prevalence in the country. To contribute to the literature and HIV policy in Cameroon, we examined the effect of mass media exposure on the uptake of HIV testing in the country. Our findings revealed that among sexually active women and men in Cameroon, the level of HIV testing was generally low although more women (78%) have ever tested for HIV compared to men (67%). This calls for the urgency of public health interventions to increase the uptake of HIV testing among females and males if Cameroon is to meet the UNAIDS targets of 95-95-95 by 2030. Furthermore, our findings demonstrated that frequent exposure to mass media—that is, being exposed to any type of mass media including the Internet, print media, radio, and television at least once a week—was associated with a higher likelihood of ever testing for HIV compared to those who are not at all exposed.

Our findings are consistent with earlier studies that established a positive association between people’s mass media exposure and the adoption of HIV prevention behaviors. For instance, in a meta-analysis examining the effectiveness of HIV campaigns using mass media across several countries, Lacroix et al. [26] found this to be effective as it did increase the usage of condoms and people’s HIV transmission and prevention knowledge. Similarly, in Uganda, Bago and Lompo [46] found among the youth that exposure to the range of mass media was associated with the uptake of HIV testing. Furthermore, Sano et al. [23] confirmed in Ghana that mass media exposure including print media, radio and television was associated with HIV testing for married women and men. In the specific context of Cameroon, Mankanchang [47] had documented the crucial role of the print media in the fight against HIV/AIDS by positively influencing women to test and adopt HIV prevention strategies. In a study examining the changing attitudes towards HIV testing among three generations of Cameroonian men, mass media was identified as one of the most effective ways to encourage HIV testing in the country [14]. Our findings underscore the utility of using a range of mass media sources to disseminate health information as it has been identified by Katirayi et al. [14] as an important source of health information on HIV in Cameroon.

Although not the focus of our study, we also observed the role of socioeconomic, demographic, locational and behavioral factors on the uptake of HIV testing in Cameroon. We observed that for both men and women, those with some form of educational attainment—that is primary education and secondary education or higher—were more likely to test for HIV compared to those with no education regardless of the type of mass media exposure—that is, Internet, print media, radio and television. Our findings endorse that of earlier studies that have observed the positive influence of educational attainment on HIV testing. Specifically, formal education makes people more conscious and assertive about their health and healthcare needs making them to appreciate the benefits of HIV testing relative to their counterparts with no formal education [48, 49].

In addition, women and men belonging to the wealthiest households were more likely to have tested for HIV compared to those in the poorest households. In a multi-country study in SSA, Ante-Testard et al. [50] also made a similar observation. In the settings of SSA including Cameroon, this finding may not be too surprising. This is because although HIV testing may be free, accessing HIV testing services may have hidden cost including paying for transportation to healthcare facilities which may work to discourage the poor from testing [51]. In Cameroon, Hadish et al. [52] also revealed that HIV testing among the youth tends to increase with increasing household wealth. Furthermore, the wealthiest tend to have the highest interaction with healthcare spaces where they can easily test for HIV [53]. Similarly, it was observed among women and men that those that were employed were more likely to have ever tested for HIV relative to their unemployed counterparts. This finding which is consistent with a systematic review by Maulsby et al. [54] may be explained by the fact that the employed are not only better placed to pay for HIV testing related cost, but they may also have an opportunity to test during health outreach programs targeted at their work sites.

We also observed that those in urban areas were more likely to test for HIV compared to their counterparts residing in rural areas. This finding sheds more light on the urban bias in the healthcare infrastructure in SSA where those in urban areas have improved access to healthcare services including HIV testing [55, 56]. In an earlier study in Cameroon, Sande et al. [51] discussed how rural residents faced additional barriers accessing HIV testing services compared to their urban counterparts who have easy access to these services. In addition, increasing age was associated with a higher likelihood of HIV testing except for women aged 60 to 64 years, and this is largely consistent with the literature [57]. Unpacking this finding among men in East Africa, Adugna and Work [58] posit that older age may be associated with increased knowledge of HIV transmission as well as perceived risk of HIV infection.

HIV behavioral attributes of women and men including having an adequate knowledge about HIV transmission and not holding HIV stigma were associated with a higher likelihood of HIV testing. These findings which are consistent with the literature [59–61], emphasize the need to strategically improve people’s knowledge of HIV transmission and reducing their HIV stigma as conduit to increasing their uptake of HIV testing. Among the youth in Cameroon, Hadish et al. [52] had made a similar observation where lifelong HIV testing was found to be higher among the youth with comprehensive HIV/AIDS knowledge compared to their counterparts who do not posses adequate knowledge.

### Study limitations

Our study has some limitations. For instance, the CDHS, which is the dataset used for our study, is collected contemporaneously, meaning the findings of our study are limited to statistical association and should, therefore, be interpreted with caution. For another, people may have other reliable sources of health information, including from healthcare professionals and healthcare facilities that were not accounted for in our study. Additionally, the decision to test for HIV may go beyond exposure to mass media to include the quality and efficacy of the information received as well as access to HIV testing services. The construct of our dependent variable, ‘ever tested for HIV,’ could have also missed the nuances of the effect of mass media exposure on more recent HIV tests. Additionally, in our study, mass media exposure was not measured by participants’ access to HIV-specific information on the various mass media platforms but by their general content. Finally, factors such as contraception and other health perceptions that could potentially influence the uptake of HIV testing were not included in our study. It would be useful to conduct a follow-up qualitative study that examines the depth of the issues contributing to the decision to test for HIV in Cameroon. Finally, our findings may suffer from response bias given the sensitivity of the topic of HIV and HIV testing in Cameroon. Despite these limitations, our study makes an important contribution to the literature and HIV policy in Cameroon, and it is among the first to examine the influence of mass media exposure on the uptake of HIV testing in the country.

## Conclusion

Our study has established that mass media exposure is associated with the uptake of HIV testing in Cameroon. In the settings of SSA, including Cameroon, evidence points to the challenges and limitations of reaching all population segments with health information through mediums such as outreach by health professionals. Given the observed association and the widespread deployment of mass media to areas that are unreachable through the traditional modes of health information, it is critical to consider mass media platforms as useful mediums that provide health information on HIV to citizens. It is particularly important not just to reach rural residents with health and HIV information through mass media, but to make provisions through the improvement and expansion of healthcare infrastructure so that they can have the opportunity to test for HIV. Overall, it may be critical to design HIV policy interventions that specifically target those with no formal education, the poor and unemployed in Cameroon.

## References

[1] Bekolo CE, Kouanfack C, Ateudjieu J, Bechem E, Ndeso S, Tendengfor N, et al. The declining trend in HIV prevalence from population-based surveys in Cameroon between 2004 and 2018: myth or reality in the universal test and treat era? BMC Public Health. 2023;23: 1–9. doi: 10.1186/s12889-023-15374-8 36915039 PMC10009959

[2] UNAIDS. Country factsheets: Haiti 2022. UNAIDS. 2021. Available: https://aidsinfo.unaids.org/%250D

[3] UNAIDS. THE PATH THAT ENDS AIDS: 2023 UNAIDS GLOBAL AIDS UPDATE. Geneva; 2023.

[4] CIA.gov. HIV/AIDS–adult prevalence rate. In: cia.gov [Internet]. 2023 [cited 7 Sep 2023]. Available: https://www.cia.gov/the-world-factbook/about/archives/2021/field/hiv-aids-adult-prevalence-rate/country-comparison

[5] Chamie G, Napierala S, Agot K, Thirumurthy H. HIV testing approaches to reach the first UNAIDS 95% target in sub-Saharan Africa. Lancet HIV. 2021;8: e225–e236. doi: 10.1016/S2352-3018(21)00023-0 33794183

[6] WHO. Cameroon making progress in the fight against HIV. In: Brazzaville [Internet]. 2023 [cited 28 Dec 2023]. Available: https://www.afro.who.int/countries/cameroon/news/cameroon-making-progress-fight-against-hiv

[7] Ministry of Health (MOH). Cameroon Population-based HIV Impact Assessment. Yaounde; 2020. Available: https://www.minsante.cm/site/?q=en

[8] CAMPHIA_Cameroon. Cameroon Population-Based Hiv Impact Assessment Camphia 2017. ICAP Columbia. 2018; 1–6.

[9] PEPFAR. THE SUSTAINABLE FINANCING INITIATIVE IN NIGERIA AT A GLANCE How SFI Contributes to a Sustainable HIV Response Spotlight: Making Health Care Accessible. 2020.

[10] Cameroon Ministry of Public Health. Cameroon’s National Strategic Plan for Fight against HIV/AIDS and STIs, 2021–2023. Gov Cameroon. Yaounde; 2021.

[11] Bonnel R. User Fees and Access to HIV/AIDS Services in Cameroon. 2019.

[12] Bousmah M al Q, Nishimwe ML, Kuaban C, Boyer S. Free access to antiretroviral treatment and protection against the risk of catastrophic health expenditure in people living with HIV: evidence from Cameroon. BMC Health Serv Res. 2021;21: 1–7. doi: 10.1186/s12913-021-06331-5 33827564 PMC8028721

[13] Ouédraogo PS. Fighting HIV/AIDS in Cameroon: A plea for a reduction in household costs. In: PH4 [Internet]. 2021 [cited 28 Jun 2023]. Available: https://p4h.world/en/news/fighting-hiv-aids-in-cameroon-a-plea-for-a-reduction-in-household-costs/

[14] Katirayi L, Tchendjou P, Tchounga B, Mbunka M, Wicks M, Conserve DF. Changing attitudes towards HIV testing and treatment among three generations of men in Cameroon: a qualitative analysis using the Fogg Behavior Model. BMC Public Health. 2023;23: 1–10. doi: 10.1186/s12889-023-15139-3 36922812 PMC10015680

[15] Ngangue P, Gagnon MP, Bedard E. Challenges in the delivery of public HIV testing and counselling (HTC) in Douala, Cameroon: Providers perspectives and implications on quality of HTC services. BMC Int Health Hum Rights. 2017;17: 1–9. doi: 10.1186/s12914-017-0118-2 28390398 PMC5385024

[16] PEPFAR. Cameroon: Country operational plan (COP) 2021. Strategic direction summary. Washington DC; 2021.

[17] Gueler A, Vanobberghen F, Rice B, Egger M, Mugglin C. The HIV Care Cascade from HIV diagnosis to viral suppression in sub-Saharan Africa: A systematic review and meta-regression analysis protocol. Syst Rev. 2017;6: 1–6. doi: 10.1186/s13643-017-0562-z 28841910 PMC5574086

[18] Akonumbo AN. AFRICAN HUMAN RIGHTS LAW JOURNAL HIV/AIDS law and policy in Cameroon: Overview and challenges. AFRICAN Hum RIGHTS LAW J. 2006;721: 85–122.

[19] Mbanya D, Sama M, Tchounwou P. Current status of HIV/AIDS in Cameroon: How effective are control strategies? Int J Environ Res Public Health. 2008;5: 378–383. doi: 10.3390/ijerph5050378 19151432 PMC3699997

[20] PEPFAR. Cameroun Country Operational Plan (COP) Strategic Direction Summary. 2022. Available: http://www.pepfar.gov/documents/organization/250290.pdf

[21] Muluh H, Nukwa Q. The challenge of HIVAIDS: The Role of the Cameroon. Int J Commun. 2007;17: 103–116.

[22] Wantchami NL. Media Performance in the Coverage of HIV/AIDS: in Four Regions of Cameroon. LAP LAMBERT Academic Publishing; 2012.

[23] Sano Y, Sedziafa a P, Amoyaw J a, Boateng GO, Kuuire VZ, Boamah S, et al. Exploring the linkage between exposure to mass media and HIV testing among married women and men in Ghana. AIDS Care. 2016;0121: 1–5. doi: 10.1080/09540121.2015.1131970 26753839

[24] Onsomu EO, Moore DK, Abuya BA, Valentine P, Duren-Winfield V. Importance of the media in scaling-up HIV testing in Kenya. SAGE Open. 2013;3: 1–12. doi: 10.1177/2158244013497721

[25] Somefun OD, Wandera SO, Odimegwu C. Media Exposure and HIV Testing Among Youth in Sub-Saharan Africa: Evidence From Demographic and Health Surveys (DHS). SAGE Open. 2019;9. doi: 10.1177/2158244019851551

[26] Lacroix JM, Snyder LB, Huedo-Medina TB, Johnson BT. Effectiveness of mass media interventions for HIV prevention, 1986–2013: A meta-analysis. J Acquir Immune Defic Syndr. 2014;66: 329–340. doi: 10.1097/QAI.0000000000000230 25007204

[27] Kansanga MM, Braimah JA, Antabe R, Sano Y, Kyeremeh E, Luginaah I. Examining the association between exposure to mass media and health insurance enrolment in Ghana. Int J Health Plann Manage. 2018;33: e531–e540. doi: 10.1002/hpm.2505 29431230

[28] Konkor I, Sano Y, Antabe R, Kansanga M, Luginaah I. Exposure to mass media family planning messages among post-delivery women in Nigeria: testing the structural influence model of health communication. Eur J Contracept Reprod Heal Care. 2019;24: 18–23. doi: 10.1080/13625187.2018.1563679 30747544

[29] Gashu KD, Yismaw AE, Gessesse DN, Yismaw YE. Factors associated with women’s exposure to mass media for Health Care Information in Ethiopia. A case-control study. Clin Epidemiol Glob Heal. 2021;12: 100833. doi: 10.1016/j.cegh.2021.100833

[30] Anfaara FW, Antabe R, Sano Y. Postpartum Married Women’s Mass Media Exposure to Family Planning Messages in the Democratic Republic of Congo: The Role of Women’s Household Decision-Making Autonomy. Health Geography in Sub-Saharan Africa: Development-Health Nexus. 2023.

[31] Sserwanja Q, Mutisya LM, Musaba MW. Exposure to different types of mass media and timing of antenatal care initiation: insights from the 2016 Uganda Demographic and Health Survey. BMC Womens Health. 2022;22: 1–8. doi: 10.1186/s12905-022-01594-4 35012537 PMC8751065

[32] Li J, Chen J. Media exposure, trustworthiness of sources and the health information literacy knowledge gap: a study in China. Health Promot Int. 2023;38: 1–12. doi: 10.1093/heapro/daad129 37837409

[33] Jiang MM, Xiao YW, Liao ZL. Pathways of Media Contact to Health Literacy in Middle-Aged and Older People: The Chain Mediation Effect of Perceived Social Support and Self-Efficacy. J Multidiscip Healthc. 2024;17: 111–121. doi: 10.2147/JMDH.S448223 38205129 PMC10778256

[34] Rikard R V, Head R, Thompson MS. Know your status: Health literacy, self-efficacy & HIV testing attitudes. J Behav Soc Sci. 2016. Available: https://www.researchgate.net/profile/RV_Rikard/publication/303382990_Know_Your_Status_Health_Literacy_Self-Efficacy_HIV_Testing_Attitudes/links/573f7cbf08ae298602e8f535.pdf

[35] Aghaji A, Burchett HED, Hameed S, Gilbert C. Assessing the capacity of primary health care facilities in Nigeria to deliver eye health promotion: Results of a mixed-methods feasibility study. PLOS Glob Public Heal. 2022;2: e0000645. doi: 10.1371/journal.pgph.0000645 36962620 PMC10022001

[36] Anasi SNI. Access to and Dissemination of Health Information in Africa: The Patient and the Public. J Hosp Librariansh. 2012;12: 120–134. doi: 10.1080/15323269.2012.666647

[37] Azevedo MJ. Historical Perspectives on the State of Health and Health Systems in Africa, Volume II. Historical Perspectives on the State of Health and Health Systems in Africa, Volume II. 2017. doi: 10.1007/978-3-319-32564-4

[38] Atuoye KN, Dixon J, Rishworth A, Galaa SZ, Boamah SA, Luginaah I. Can she make it? Transportation barriers to accessing maternal and child health care services in rural Ghana. BMC Health Serv Res. 2015;15: 1–10. doi: 10.1186/s12913-015-1005-y 26290436 PMC4545969

[39] Antabe R, Kansanga M, Sano Y, Kyeremeh E, Galaa Y. Utilization of breast cancer screening in Kenya: what are the determinants? BMC Health Serv Res. 2020;2: 1–9. 10.1186/s12913-020-5073-2 PMC7079358 32183801

[40] Mwini-Nyaledzigbor PP, Agana AA, Pilkington FB. Lived Experiences of Ghanaian Women With Obstetric Fistula. Health Care Women Int. 2013;34: 440–460. doi: 10.1080/07399332.2012.755981 23641897

[41] National Institute of Statistics (Cameroon) and ICF. 2018 Cameroon Demographic and Health Survey Summary Report. Rockville, Maryland, USA NIS ICF. 2020; 1–20.

[42] Amoak D, Osei-kye N, Anfaara FW, Sano Y, Luginaah I, Amoak D, et al. Understanding the uptake of HIV testing among women in Liberia: the role of female genital mutilation / cutting Understanding the uptake of HIV testing among women in Liberia: the role of female genital mutilation / cutting. African J AIDS. 2023;22: 226–236. doi: 10.2989/16085906.2023.2275695 38015895

[43] Luginaah NA, Konkor I, Lawson ES, Mkandawire P, Husbands W, Omorodion F, et al. Concurrent sexual partnerships and HIV testing among heterosexual Black men in Ontario, Canada: findings from the weSpeak study. Ethn Health. 2021;0: 1–16. doi: 10.1080/13557858.2021.1976395 34494926

[44] Wandera SO, Kwagala B, Maniragaba F. Prevalence and determinants of recent HIV testing among older persons in rural Uganda: A cross-sectional study. BMC Public Health. 2020;20: 1–10. doi: 10.1186/s12889-020-8193-z 32005198 PMC6995239

[45] StataCorp. Stata Survey Data Reference Manual. Stata Press Publication. A Stata Press Publication StataCorp LLC College Station, Texas. Texas: StataCorp LP; 2021. Available: https://www.stata.com/manuals/svy.pdf

[46] Bago JL, Lompo ML. Exploring the linkage between exposure to mass media and HIV awareness among adolescents in Uganda. Sex Reprod Healthc. 2019;21: 1–8. doi: 10.1016/j.srhc.2019.04.004 31395226

[47] Mankanchang SE. THE ROLE OF THE PRINT MEDIA IN THE FIGHT AGAINST HIV/AIDS AMONGST AMONGST THE WOMEN IN CAMEROON: CASE OF THE CAMEROON POST NEWSPAPER. Lund University. Lund University. 2010.

[48] Sambah F, Baatiema L, Appiah F, Ameyaw EK, Budu E, Ahinkorah BO, et al. Educational attainment and HIV testing and counselling service utilisation during antenatal care in Ghana: Analysis of demographic and health surveys. PLoS One. 2020;15: 1–11. doi: 10.1371/journal.pone.0227576 31940331 PMC6961934

[49] Muyunda B, Musonda P, Mee P, Todd J, Michelo C. Educational Attainment as a Predictor of HIV Testing Uptake Among Women of Child-Bearing Age: Analysis of 2014 Demographic and Health Survey in Zambia. Front Public Heal. 2018;6: 1–9. doi: 10.3389/fpubh.2018.00192 30155454 PMC6102411

[50] Ante-Testard PA, Benmarhnia T, Bekelynck A, Baggaley R, Ouattara E, Temime L, et al. Temporal trends in socioeconomic inequalities in HIV testing: an analysis of cross-sectional surveys from 16 sub-Saharan African countries. Lancet Glob Heal. 2020;8: e808–e818. doi: 10.1016/S2214-109X(20)30108-X 32446346

[51] Sande L, Maheswaran H, Mangenah C, Mwenge L, Indravudh P, Mkandawire P, et al. Costs of accessing HIV testing services among rural Malawi communities. AIDS Care—Psychol Socio-Medical Asp AIDS/HIV. 2018;30: 27–36. doi: 10.1080/09540121.2018.1479032

[52] Hadish MT, Mao J, Gong G, Hadish BT, Tesfamariam EH, Tesfay AW, et al. Predictors of Health-Seeking Behavior: HIV Test Experiences among Youth Aged 15–24 Years in Cameroon and Gabon. J Transm Dis Immun. 2017;01. doi: 10.21767/2573-0320.100010

[53] Deane K, Wamoyi J, Mgunga S, Changalucha J. HIV testing attitudes and practices amongst “wealthy men”: qualitative evidence from Tanzania. Cult Heal Sex. 2022;24: 1215–1229. doi: 10.1080/13691058.2021.1941261 34254898

[54] Maulsby CM, Ratnayake A, Hesson D, Mugavero MJ, Latkin CA. A Scoping Review of Employment and HIV Catherine. AIDS Behav. 2020;24: 2942–2955. doi: 10.1007/s10461-020-02845-x.A 32246357 PMC7716244

[55] Antabe R, Luginaah NA, Kangmennaang J, Mkandawire P. Determinants of cervical cancer screening among women living with HIV in Zimbabwe. Health Promot Int. 2023;38: 1–12. doi: 10.1093/heapro/daad073 37440254

[56] Amoak D, Konkor I, Mohammed K, Saaka SA, Antabe R. Exposure to mass media family planning messages among men in Nigeria: analysis of the Demographic and Health Survey data. PeerJ. 2023;11: 1–16. doi: 10.7717/peerj.15391 37273544 PMC10237178

[57] Worku MG, Teshale AB, Tesema GA. Prevalence and associated factors of HIV testing among young (15–24) women in eastern Africa: a multilevel analysis of demographic health survey data (2008–2018). Arch Public Heal. 2022;80: 1–8. doi: 10.1186/s13690-022-00879-2 35410302 PMC9004117

[58] Adugna DG, Worku MG. HIV testing and associated factors among men (15–64 years) in Eastern Africa: a multilevel analysis using the recent demographic and health survey. BMC Public Health. 2022;22: 1–9. doi: 10.1186/s12889-022-14588-6 36434555 PMC9701050

[59] Kuehne A, Koschollek C, Santos-Hövener C, Thorlie A, Müllerschön J, Tshibadi CM, et al. Impact of HIV knowledge and stigma on the uptake of HIV testing–Results from a community-based participatory research survey among migrants from sub-Saharan Africa in Germany. PLoS One. 2018;13: 1–19. doi: 10.1371/journal.pone.0194244 29641527 PMC5894987

[60] Chimoyi L, Tshuma N, Muloongo K, Setswe G, Sarfo B, Nyasulu PS. HIV-related knowledge, perceptions, attitudes, and utilisation of HIV counselling and testing: A venue-based intercept commuter population survey in the inner city of Johannesburg, South Africa. Glob Health Action. 2015;8. doi: 10.3402/gha.v8.26950 25925192 PMC4414782

[61] Budu E, Seidu AA, Armah-Ansah EK, Mohammed A, Adu C, Ameyaw EK, et al. What has comprehensive HIV/AIDS knowledge got to do with HIV testing among men in Kenya and Mozambique? Evidence from Demographic and Health Surveys. J Biosoc Sci. 2021;2016: 558–571. doi: 10.1017/S0021932021000237 34099074

